# The correlated expression of immune and energy metabolism related genes in the response to *Salmonella enterica* serovar Enteritidis inoculation in chicken

**DOI:** 10.1186/s12917-020-02474-5

**Published:** 2020-07-25

**Authors:** Yuanmei Wang, Xiuxiu Miao, Huilong Li, Pengcheng Su, Lili Lin, Liying Liu, Xianyao Li

**Affiliations:** 1 Shandong Provincial Key Laboratory of Animal Biotechnology and Disease Control and Prevention, College of Animal Science and Veterinary Medicine, Shandong Agricultural University, 271018 Taian, China; 2 Present Address: Current affiliation: Key Laboratory of Animal (Poultry) Genetics Breeding and Reproduction, Ministry of Agriculture, Institute of Animal Science, Chinese Academy of Agricultural Sciences, 100193 Beijing, China; 3 College of Life Science, Shandong Agricultural University, 271018 Taian, China

**Keywords:** *Salmonella enterica* serovar Enteritidis, Chicken, Energy metabolism, Immune, Gene expression

## Abstract

**Background:**

*Salmonella enterica* serovar Enteritidis (SE) is one of the food-borne pathogenic bacteria, which affects poultry production and poses severe threat to human health. The correlation of immune system and metabolism in chicken after SE inoculation is important but not clear. In the current study, we identified the expression of immune and energy metabolism related genes using quantitative PCR to evaluate the correlation between immune system and energy metabolism against SE inoculation in Jining Bairi chicken.

**Results:**

ATP5G1, ATP5G3 and ND2 were significantly up-regulated at 1 dpi (day post inoculation), and ATP5E, ATP5G1, ATP5G3 were significantly down-regulated at 7 dpi (*P* < 0.05). IL-8 and IL-1*β* were significantly down-regulated at 1 dpi, IL-8 and IL-18 were significantly down-regulated at 3 dpi, IL-8 and BCL10 were significantly up-regulated at 7 dpi (*P* < 0.05).

**Conclusions:**

These findings indicate that the correlation between immune and energy metabolism related genes gradually change with time points post SE inoculation, from one homeostasis to an opposite homeostasis with 3 dpi as a turning point. These results will pave the foundation for the relationship between immune system and energy metabolism in the response to SE inoculation in chicken.

## **Background**

*Salmonella enterica* serovar Enteritidis (*S*. Enteritidis) is frequently associated with food-borne disease in the world which can infect a wide range of hosts and frequently reach the human food chain causing food-borne disease [1]. In China, Salmonella causes 70–80% of foodborne bacterial outbreaks and the primary vehicles of transmission are raw or undercooked chicken [2]. *Salmonella* is estimated to be responsible for approximate 1 million cases of illness and more than 450 deaths annually in the United States [3]. Poultry is considered as a primary source of foodborne diseases, especially *Salmonella* Enteritidis infection. *S*. Enteritidis is responsible for 36% of *Salmonella* outbreaks in the United States [4]. The losses caused by egg-related salmonellosis in Australia have reached $44 million annually [5]. *S*. Enteritidis causes significant economic losses to the poultry industry due to the mortality, nutrient malabsorption, retarded growth rate, and decreased egg production [1].

The response to *S*. Enteritidis infection is regulated through altering gene expression. Many genes have been disclosed in the response to *S*. Enteritidis infection [6–8]. Toll-like receptor (TLR)-mediated recognition of pathogens is now thought to have a crucial role in innate and adaptive immune [9]. Subsets of immune and inflammatory cells interact via interleukins (ILs) and IFNs [10]. TLRs and ILs have been reported to be associated with *S.* Enteritidis infection in chicken [11–15]. It has been reported that complex networks that intimately connect metabolism and immunity in human [16]. Wu et al. proved that the complicated interaction between the immune system and metabolism contributes to the immune responses to SE inoculation of layers at 14 dpi at the onset of lay [17].

Metabolism, a series of orderly chemical reactions, controls production, maintenance, destruction of biomolecules, and distribution of energy to organisms [16]. Energy metabolism is the process of generating energy (ATP) from nutrients. Mitochondria are the primary energy-generating system in most eukaryotic cells and participate in intermediary metabolism, oxidative phosphorylation, calcium signaling, and apoptosis [18]. Genes from the mitochondrial respiratory chain complex gene family provide instructions for proteins involved in oxidative phosphorylation. Five protein complexes (I-V) are involved in this process. Mitochondrial complex V consists of 19 genes in human. The function of genes involved in complex I and V has been reported, such as mt-ND2 [19], ATP5E [20], and ATP5G3 [21, 22]. Subunit 2 of NADH dehydrogenase (ND2) is one of genes comprising of mitochondrial complex I [23], which plays a critical role in controlling the production of the mitochondrial reactive oxygen species in chicken [24]. Mt-ND2 gene was extensively expressed in tissues, and the expression was affected by dietary fat types and chicken age [19]. The altered expression of mt-ND2 is critical to multiple disease in human [25, 26]. ATP synthase epsilon subunit (ATP5E) gene was found to encode the mitochondrial F0F1 ATP synthase subunit epsilon [27]. In addition, ATP5E was proven to be required for normal spindle orientation during embryonic divisions in Drosophila [28]. ATP5G1, a key component of complex V of the oxidative phosphorylation chain, encodes a subunit of mitochondrial ATP synthase and catalyzes ATP synthesis [29] and is involved in the biological process of oxidative phosphorylation and might be associated with oxidative stress [30]. The expression of ATP5G1 and ATP5G3 is actively regulated in response to various physiological stimuli, and maintain the basal levels of the c-Fo subunit [31]. However, little information is available on the regulation of *S*. Enteritidis inoculation of energy metabolism related genes and their correlation with immune related genes.

In this study, we aim to investigate the correlation between immunity and energy metabolism in chicken in the response to SE inoculation. The mRNA expression of immune and energy metabolism related genes after SE inoculation in chicken spleen will be analyzed.

## **Results**

### The expression of energy metabolism related genes in spleen

**Fig. 1:**
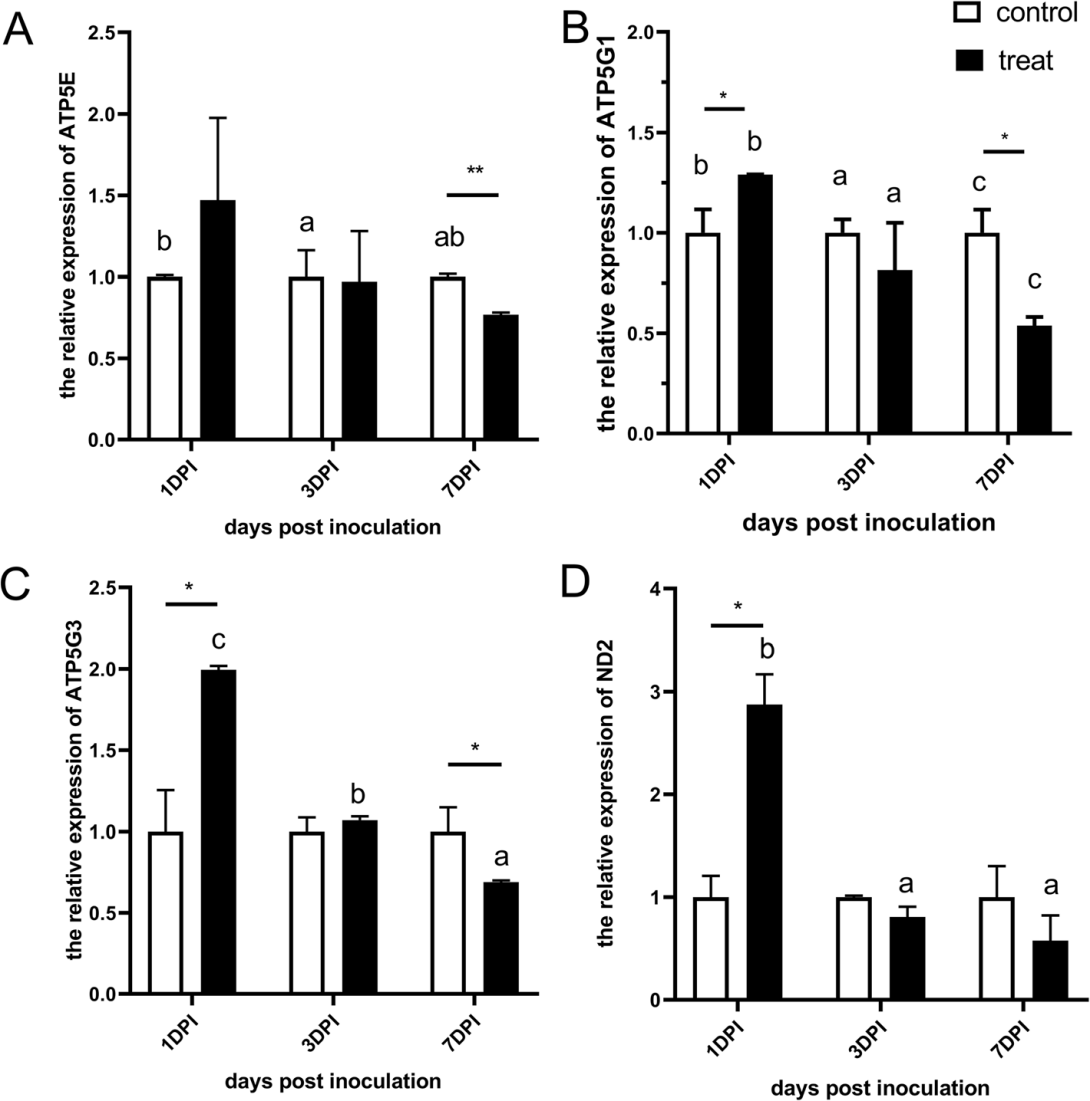
Relative mRNA expression of energy metabolism related genes in spleen between control and treat chickens. The relative expression was normalized with the control at the same time point following SE inoculation. * indicated *P* < 0.05, ** indicated *P* < 0.01. Different letters within each group indicate statistical difference (*P* < 0.05)

The expression of ATP5E, ATP5G1, ATP5G3 and ND2 in control and treat group was shown in Fig. 1. At 1 dpi, the expression level of ATP5G1, ATP5G3 and ND2 in the treat group was significantly higher than that in the control group with fold change of 1.29, 1.99 and 2.87, respectively (*P* < 0.05) (Fig. 1b, c and d). At 7dpi, the expression of ATP5E, ATP5G1 and ATP5G3 in the treat group was significantly lower than that in the control group with a fold change of 0.77, 0.54 and 0.69, respectively (*P* < 0.05) (Fig. 1a, b and c).

Within the treat group, ATP5G1 and ATP5G3 were significantly decreased across the different time points (*P* < 0.05) (Fig. 1b and c). The expression of ATP5G1 and ATP5G3 was significantly higher at 3 dpi compared to that at 7 dpi (*P* < 0.05) (Fig. 1b and c). Within the control group, the expression of ATP5E and ATP5G1 was significantly higher at 3 dpi than that at 1 dpi (*P* < 0.05) (Fig. 1a and b). The expression of ATP5G1 at 7 dpi was the lowest across 3 different time points (*P* < 0.05) (Fig. 1b).

### The expression of immune-related genes

**Fig. 2:**
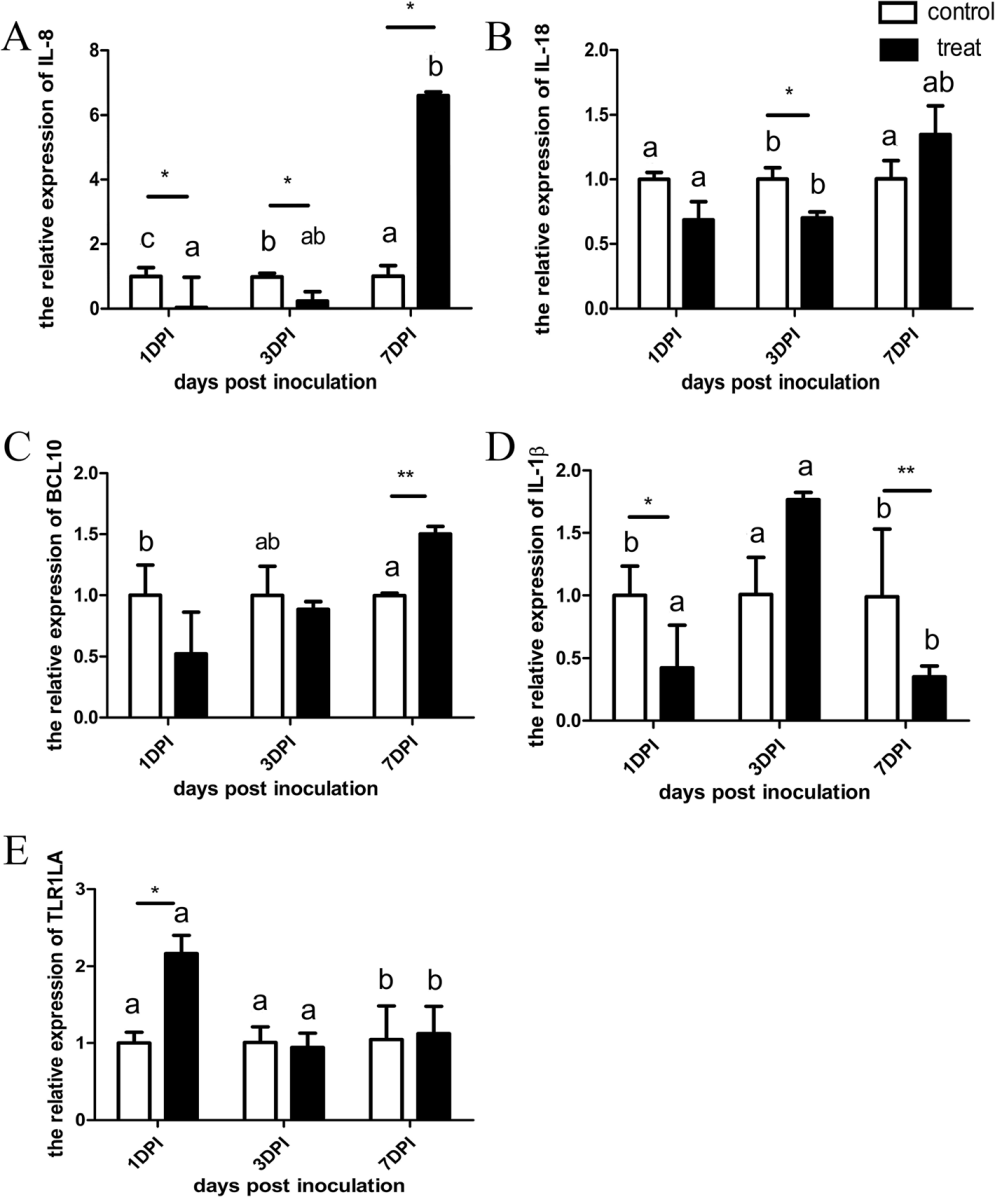
Relative mRNA expression of immune-related genes in spleen between control and treat chickens. The relative expression was normalized with the control at the same time point following SE inoculation. *indicated *P* < 0.05, ∗∗ indicated *P* < 0.01. IL = interleukin. Different letters within each group indicate statistical difference (*P* < 0.05)

The expression of immune-related genes was shown in Fig. 2. At 1 and 3 dpi, the expression of IL-8 in the treat group was significantly lower compared with that in the control group with a fold change of 0.03 and 0.23, respectively (*P* < 0.05) (Fig. 2a). At 3 dpi, the expression of IL-18 in the treat group was significantly lower compared with that in the control group (*P* < 0.05) (Fig. 2b). At 7 dpi, the expression of IL-8 and BCL10 in the treat group was significantly higher compared with that in the control group with a fold change of 6.56 and 1.50, respectively (*P* < 0.05) (Fig. 2a and c). At 1 and 7 dpi, the expression of IL-1*β* in the treat group was significantly lower compared with that in the control group with a fold change of 0.42 and 0.35, respectively (*P* < 0.05) (Fig. 2d). At 1 dpi, the expression of TLR1LA in the treat group was significantly higher compared with that in the control group with a fold change of 2.17 (*P* < 0.01) (Fig. 2e).

Within the treat group, IL-8 at 1 dpi was significantly lower compared to that at 7 dpi (*P* < 0.05) (Fig. 2a). IL-1*β* at 7 dpi was significantly lower compared to that at 1 and 3 dpi (*P* < 0.05) (Fig. 2d). TLR1LA at 1 dpi was significantly higher compared to that at 7 dpi (*P* < 0.05) (Fig. 2e).

Within the control group, IL-18 at 3 dpi was significantly higher compared to that at 1 and 7dpi (*P* < 0.05) (Fig. 2b). BCL10 at 1 dpi was significantly higher compared to that at 7 dpi (*P* < 0.05) (Fig. 2c). IL-1*β* at 3 dpi was significantly higher compared to that at 1 and 7 dpi (*P* < 0.05) (Fig. 2d). TLR1LA at 1 dpi was significantly lower than that at 3 and 7 dpi (*P* < 0.05) (Fig. 2e).

### Correlative expression of immune-related and energy metabolism related genes following SE inoculation

**Fig. 3:**
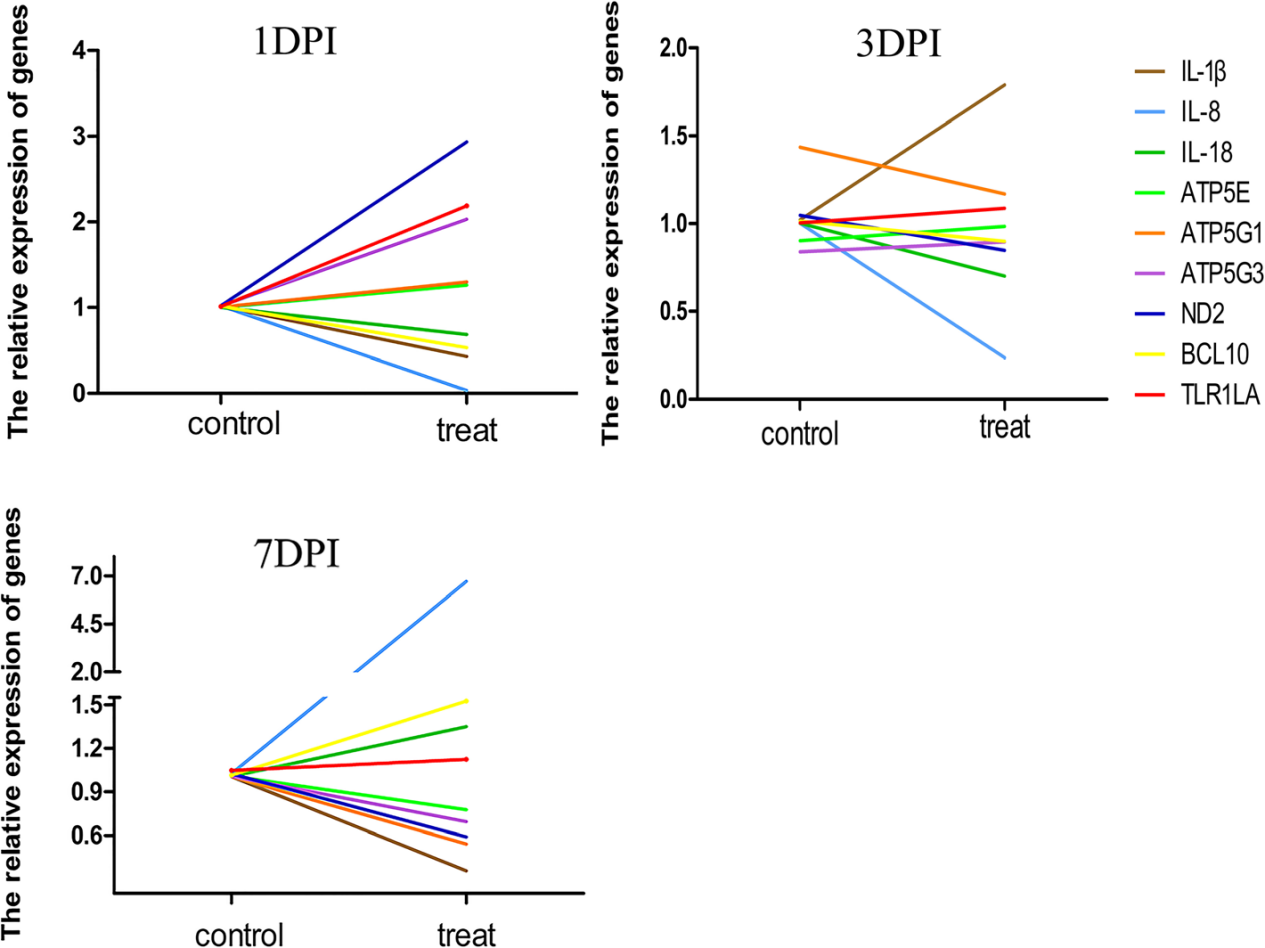
Correlative expression between immune and energy metabolism related genes

At 1 dpi, the expression of ATP5E, ATP5G1, ATP5G3 and ND2 in the treat group was higher than that in the control group, however, the expression of IL-1*β*, IL-8, IL-18 and BCL10 in the treat group was lower than that in the control group (Fig. 3). At 3 dpi, the expression of ATP5E, ATP5G3, TLR1A and IL-1*β* was higher while the expression of ATP5G1, ND2, IL-8, IL-18 and BCL10 was lower in the treat group compared with that in the control group (Fig. 3). At 7 dpi, with respect to the control group, the expression of ATP5E, ATP5G1, ATP5G3 and ND2 was lower in the treat group, while the expression of IL-8, IL-18, BCL10 and TLR1LA was higher in the treat group (Fig. 3).

## **Discussion**

The correlative expression of metabolism related genes and immune related genes has been studied in the current study. The mitochondrial ATP synthase activity plays a key role in energy production in mammals [32]. ATP5G1, ATP5G2 and ATP5G3 encode the mitochondrial ATP synthase c-Fo subunit [33]. ATP5G1 gene expression is actively regulated in response to various physiological stimuli (such as ontogenic development and cold acclimation), whereas the ATP5G2 and ATP5G3 maintain the basal levels of the c-Fo subunit [33, 34]. However, few studies focused on the energy metabolism in chicken inoculated with SE. In the current study, we found SE inoculation altered the expression of four energy metabolism related genes at different stages. Knockdown of ATP5G1 results in reduced ATP levels and oxidative phosphorylation activity, and increases the amount of reactive oxygen species [35]. It has been reported that ATP5G1 involves in immune response and impaired immunoproteasome assembly via PA28–20S-PA28 and PA700–20S-PA28 complexes [36–39]. The up-regulated ATP5G3 gene switches the Warburg effect to oxidative phosphorylation with slowing energy production rate and inhibiting cancer cells growth [40]. At day 1 post SE inoculation, the expression ATP5G1 36 and ATP5G3 was up-regulated in the current study (*P* < 0.05). Mitochondria may be coping with the SE infection through up-regulated energy metabolism related gens at the early period of inoculation. The down-regulation of ATP5E is directly related with mitochondrial injury, and leads to less energy production [41]. Additionally, ATP5E, ATP5G1, ATP5G3 and ATP5E were down-regulated in papillary thyroid carcinoma (PTC) patient and ATP5E was highly associated to PTC diagnosis [42]. In the current study, ATP5G1 (0.77-fold), ATP5G3 (0.54-fold) and ATP5E (0.6-fold) were down-regulated at 7 days post SE inoculation which indicates the SE inoculation lead to mitochondrial injury and reduce energy production at later period of inoculation.

Toll-like receptors are a family of germ line-encoded pattern recognition receptors which activate rapid inflammatory responses upon detection of their cognate ligands [43]. TLR1LA is needed for the recognition of bacterial lipoprotein and LPS and is widely expressed among chicken tissues [44, 45]. In chickens, TLR1.1 and TLR1.2, two members of the TLR1/TLR6/TLR10 family, can recognize lipoproteins and peptidoglycan, respectively [46, 47]. TLR1 was significantly up-regulated in chicken intestine at 2 and 4 days post H9N2 influenza virus [48]. It has been reported that TLR1LA was significantly up-regulated in the cecum (3-fold) and the ileum (2.6-fold) at 24 h post SE infection in 2-day-old chickens [49]. A similar increase in TLR1LA (2.7-fold) was also observed in chicken spleen at 1 day post SE inoculation in the current study. The data demonstrate that increased expression of TLR1LA in various tissues may play a role in facilitating increased recognition of lipoproteins and peptidoglycan.

BCL10 is an intracellular signaling protein which positively regulates lymphocyte proliferation by connecting antigen receptor induced signals of B and T cells to the activation of the transcription factor NF-κB [50, 51]. Interleukin 8 (IL-8) is the major chemoattractant and activator for neutrophils and a key component of innate immunity [52]. BCL10 was significantly down-regulated in cecum at 7 days post SE inoculation in White Leghorn [53]. In the current study, SE inoculation induced both BCL10 (1.5-fold) and IL-8 (6.56-fold) in spleen at 7 dpi. The increased expression of immune-related genes would facilitate limiting pathogens further proliferation. IL-8 and IL-1*β* are two major pro-inflammatory cytokines which play a central role in initiation of inflammatory responses against bacterial-and viral-infections [54]. In the current study, IL-1*β* was up-regulated at 3dpi, which was supported by the expression in chicken cecum following SE inoculation [15]. IL-8 and IL-18 in cecum were significantly increased at 3 days following *S.* Enteritidis infection [15], which was inconsistent with our study probably due to different genetic background and tissues. IL-18 provides an important link between the innate and adaptive immune responses [55] and is associated with immune organ damage of Avian reovirus and immune suppression [56]. BCL10 is a mediator of LPS-induced activation of IL-8 in human intestinal epithelial cells [57]. These results indicated SE inoculation could be regulated by BCL10 through mediating IL-8 at 7 dpi and induced immune damage in chicken spleen.

The immune responses are highly energy dependent process and energy metabolism is also involved in the immune networking for self-defense and against pathophysiology [58]. Various stresses may increase energy demands for immune regulation at the expense of energy reserves, and then reduce the energy available for growth and other processes [59, 60]. Furthermore, disease and nutrition are closely interlinked [61]. The immune variations under ambient pressures may trigger energy metabolic changes in mollusks [59, 62]. It has been reported that interaction between the immune system and metabolism contributes to the immune responses of egg-type chicken to SE inoculation at the onset of lay [17]. In the current results, IL-8 was down-and up- regulated at 1 and 7 days post SE inoculation. ATP5G1 and ATP5G3 were up-and down-regulated at 1 and 7 days post SE inoculation. These results suggested there existed a negative correlation between IL-8 and ATP5G1, ATP5G3 at 1 and 7 days post SE inoculation. The correlation between immune related genes and energy metabolism related genes was multidirectional at 3 dpi. These findings indicated that the correlation between immune and energy metabolism related genes gradually change with time points post SE inoculation, from one homeostasis to an opposite homeostasis. Day 3 post SE inoculation maybe a key point to regulate the correlation between immune system and energy metabolism in the response to SE inoculation. The findings in the current study would deepen the understanding of comprehensive interaction between immune system and energy metabolism in the response to SE inoculation in chicken.

## **Conclusions**

These findings indicated that the correlation between immune and energy metabolism related genes gradually change with time points post SE inoculation, from one homeostasis to an opposite homeostasis. Day 3 post SE inoculation maybe a key point to regulate the correlation between immune system and energy metabolism in the response to SE inoculation. These results provided the foundation for the relationship between immune system and energy metabolism in the response to SE inoculation in chicken.

## **Methods**

### Animals and sample collection

Jining Bairi Chicken, a Chinese local chicken breed used in the current study was provided by Shandong Bairi Chicken Breeding Co., Ltd. (Shandong, China). The *S.* Enteritidis strain (CVCC3377) used in the current study was purchased from the China Veterinary Culture Collection Center (Beijing, China). To make the inoculant, SE were enriched in LB broth at 37℃ for 16 h, pelleted at 4,000 rpm for 5 min, and diluted with sterilized PBS. The concentration of SE in the inoculant was measured by the plating method. The animal trial was performed as described previously [6]. In brief, 168 two-day-old *S.* Enteritidis–negative chickens were randomly into 2 groups with 84 chickens in each of treat and control groups. Chickens in treat and control groups were raised in 2 separate incubators with the same environmental conditions and free access to sterilized feed and water. Each chicken in the treat group was orally inoculated with 0.3 ml of 10^9^ colony-forming units (cfu)/ml SE inoculant. Each chicken in the control group inoculated with 0.3 ml sterile phosphate buffer saline (PBS). Twelve chickens from each of the treat and control groups were euthanized by cervical dislocation for sample collection at 1, 3, 7, 14, 21, 28 and 35 days post-inoculation. Cecum content was collected for SE enumeration. The chicken mortality was zero in the treat group. Chickens in the control group were SE negative. Spleen samples were collected and stored at -80 °C for further utilization. Three individual spleen samples in the treat group with the highest bacterial number and three random individual spleen samples in the control group at each of 1, 3 and 7 dpi were used in the current study. All animal procedures were approved by Shandong Agricultural University Animal Care and Use Committee.

### Total RNA extraction and cDNA synthesis

Total RNA was extracted from each individual spleen sample using TRIzol reagent (Invitrogen, US) following the manufacturer’s instructions and stored at -80℃ until further use. In total, 18 RNA samples were used in the current study. The quantity and quality of RNA sample was evaluated using DS-11 Spectrophotometer (DeNovix, US) and gel electrophoresis, respectively. Total RNA was digested with DNase I to eliminate the genomic DNA and reverse transcribed into the cDNA using Transcriptor First Strand cDNA Synthesis Kit (Perfect Real Time) (Takara, Dalian, China) according to the manufacturer’s instructions. The 20 *µ*L reaction system was used and contained total RNA 1 *µg*, 5 × Primer Script Buffer (for Real Time) 4 *µ*L, Primer Script RT Enzyme Mix I 1 *µ*L, Random 6 mers (100 *µM*) 1 *µ*L, and adding RNase-free ddH_2_O to 20 *µ*L. The reaction condition was 37 ℃ for 15 min, 85 ℃ for 5 s.

### Quantitative real time-polymerase chain reaction (qRT-PCR)

ATP5E, ATP5G1, ATP5G3, ND2, BLC10, TLR1A, IL-1*β*, IL-8 and IL-18 were selected for qRT-PCR. Beta-actin (*β*-actin) was used as the reference gene to adjust relative quantification of the target genes. The specific primer sequences of all genes were designed using Primer Premier 5.0 and listed in Table 1. The qRT-PCR was performed using the Applied Biosystems 7500 Fast system (Applied Biosystems, US) with a SYBR®Premix Ex Taq™ Kit (Roche, US). A reaction mixture (20 *µ*L) consisted of cDNA 2 *µ*L, forward primer 0.5 *µ*L, reverse primer 0.5 *µ*L, SYBR Green Master (Mix) 10 *µ*L, and ddH_2_O 7 *µ*L. The qRT-PCR amplification condition was as follows: 50℃ for 2 min, 95℃ for 10 min, followed by 40 cycles at 95℃ for 15 s, 60 ℃ for 1 min, finally, a single melt cycle was 95℃ for 15 s and 65℃ for 1 min. Triplicate was performed for each cDNA sample to run the qRT-PCR.

**Table 1:** Primer sequences and product size

Gene Name	Accession No.	Sequence (5’-3’)	Product size(bp)
*β*-actin	NM_205518	Forward: TGCTGTGTTCCCATCTATCG Reverse: TTGGTGACAATACCGTGTTCA	150
ATP5E	XM_001231374.5	Forward: TTGTTGGGAGTGGAGTGT Reverse: TCACCATTCACAGAGCAGT	204
ATP5G1	XM_001233602.5	Forward: GGACACGGCAAGTAATAGG Reverse: CATCAAACAGAAGAGACCCA	190
ATP5G3	NM_001277855.1	Forward: AGGACTGGAGAGGGCAAAT Reverse: CCATCAGACAGAAGAGACCC	230
ND2	YP_009558653.1	Forward: ACACCTTAGCCATCATCCC Reverse: ATCAGGCGTTGGTTATGC	198
TLR1LA	NM_001007488	Forward: GCTTGACTTTAGTGCCTTCATGTTT Reverse: GCAAGCAATTGGCAGTAAGCT	142
BCL10	NM_001201487	Forward: GGACTGGATGCTTTGGTTGA Reverse: GAAGGGAGACAGACATAAAT	237
IL-1*β*	XM_015297469.1	Forward: AGAAGAAGCCTCGCCTGGAT Reverse: CCTCCGCAGCAGTTTGGT	131
IL-8	NM_205498.1	Forward: GGCTTGCTAGGGGAAATGA Reverse: AGCTGACTCTGACTAGGAAACTGT	200
IL-18	XM_015297946.2	Forward: GCTTCGGTTTTCACTGGTTCTC Reverse: ACACTCTGTCTGAGGTGCCAAA	117

### Statistical analysis

The relative quantification of target gene was determined using the 2^−ΔΔCt^ method [63]. The difference of expression level for each gene between treat and control groups within each time point was analyzed using student T-test, across different time points within treat or control group were analyzed using the general linear model procedure of SAS 8.1 (SAS Institute, Cary, NC). *P* < 0.05 was considered significant difference.

## Abbreviations

SE: *Salmonella enterica serovar Enteritidis*
BCL10: B-cell CLL/lymphoma 10
TLR: Toll-like receptor
IL: Interleukin
PBS: Phosphate buffer saline
qRT-PCR: Quantitative real time-polymerase chain reaction
*β*-actin: Beta-actin

## Availability of data and materials

The data used and/or analyzed in the current study are available from the corresponding author on reasonable request.
